# Real-world efficacy assessment for sintilimab in recurrent or metastatic cervical cancer

**DOI:** 10.7717/peerj.20477

**Published:** 2025-12-19

**Authors:** Dan Zhou, Ruiwei Wang, Lingjun Huang, Ping Yan, Kaijian Ling, Zhiqing Liang

**Affiliations:** 1 Department of Gynecology and Obstetrics, The First Affiliated Hospital (Southwest Hospital) of Army Medical University, Chongqing, China; 2 Department of Gynecology and Obstetrics, Guiqian International General Hospital, Guiyang, Guizhou, China

**Keywords:** Cervical cancer, Efficacy, Immunotherapy, PD-1 monoclonal antibodies, Safety

## Abstract

**Background:**

Programmed death 1 (PD-1) monoclonal antibodies have been reported as a first-line therapeutic option for recurrent cervical cancer, especially for programmed death ligand 1 (PD-L1)-positive tumors. However, real-world data on the use of PD-1 monoclonal antibodies in patients with an undefined PD-L1 status is scarce. This study analyzed the efficacy and safety of sintilimab in patients with recurrent or metastatic cervical cancer.

**Methods:**

Data of patients who received sintilimab for the first time in the First Affiliated Hospital of the Army Medical University from November 2019 to July 2022 were collected. The efficacy and safety of sintilimab were evaluated according to Response Evaluation Criteria in Solid Tumors version 1.1 (RECIST v1.1) and Common Terminology Criteria for Adverse Events (CTCAE) Version 5.0, respectively. The Kaplan–Meier curve and log-rank test were used to analyze patient survival.

**Results:**

Twenty-seven (27) patients were included in the study. The median follow-up was 17 (range, 6–40) months. The objective response rate (ORR) and disease control rate (DCR) were 69.6% and 87.0%, respectively, in efficacy-evaluable patients. The ORR of the combination therapy was significantly higher than that of the monotherapy (82.3% *vs.* 33.3%, *P* = 0.045). The ORR was 91.7% for patients who initiated treatment within two months and 37.5% for those who started treatment after two months (*P* = 0.018). In the intention-to-treat population, 55.5% of the patients experienced adverse events, and 14.8% of the patients had grade 3 or higher treatment-related adverse events.

**Conclusions:**

Sintilimab appeared to demonstrate satisfactory efficacy and safety in the study. The combination therapy showed a higher ORR compared to monotherapy. Furthermore, early initiation of sintilimab as first-line therapy within two months following recurrence is more effective compared to delayed initiation beyond this period in patients with recurrent disease.

## Introduction

Cervical cancer remains the fourth most prevalent malignancy among women worldwide. The global incidence and mortality rates show considerable geographic disparities. Between 1990 and 2021, both the incidence and mortality counts of cervical cancer showed a steady and consistent increase (Li et al., 2025). Early-stage cervical cancer is associated with a generally favorable prognosis, with surgery remaining the cornerstone of treatment. Recent clinical trials, including the ConCerv (Schmeler et al., 2021), LESSER (Carneiro et al., 2023), and SHAPE (Plante et al., 2024) studies, have provided robust evidence supporting conservative and fertility-preserving surgical strategies for low-risk, early-stage cervical cancer (Xu et al., 2025; D’Augè, Donato & Giannini, 2024). For advanced and recurrent metastatic cervical cancer, significant therapeutic advances have been achieved, particularly with the introduction of anti-angiogenic agents, immune checkpoint inhibitors, and antibody-drug conjugates (Francoeur, Monk & Tewari, 2025; D’Oria et al., 2024). Despite these developments, the treatment of advanced-stage and recurrent metastatic cervical cancer remains a significant clinical challenge.

Platinum-based chemotherapy has been the established standard of care for patients with recurrent or metastatic cervical cancer (Cohen et al., 2019). The preferred chemotherapy regimen for these patients is cisplatin combined with paclitaxel. For patients who have previously received cisplatin, a combination of carboplatin and paclitaxel may be a beneficial alternative. Due to the limited efficacy and severe toxicity associated with chemotherapy, the prognosis of patients with recurrent or metastatic cervical cancer remains poor, with a five-year survival rate of 17% (Marret, Borcoman & Le Tourneau, 2019). Since the Gynecologic Oncology Group (GOG) 240 trial (Tewari et al., 2017), the use of bevacizumab in combination with chemotherapy has been approved as a first-line treatment for recurrent or metastatic cervical cancer. However, the addition of bevacizumab has yielded only limited improvement in patient outcomes. Given the efficacy of current treatments, more effective strategies are urgently required to meet clinical needs.

Immunotherapy has emerged as the new standard of care for the treatment of patients with metastatic, persistent, or recurrent cervical cancer. As a form of immunotherapy, immune checkpoint inhibitors targeting the anti-programmed death 1 (PD-1)/programmed death ligand 1 (PD-L1) pathway have been widely explored in cervical cancer. The efficacy and safety of pembrolizumab, a PD-1 antibody, have gradually been established in patients with recurrent or metastatic cervical cancer. The KEYNOTE-158 study (Chung et al., 2019) reported the objective response rates (ORRs) for pembrolizumab as 12.2% in all recurrent or metastatic cervical cancer patients and 14.3% in PD-L1–positive patients who had previously received chemotherapy. However, the ORR was 0% for patients who were PD-L1 negative.

Pembrolizumab was approved in June 2018 as a second-line treatment option for recurrent or metastatic cervical cancer with a PD-L1 combined positive score (CPS) of 1 or higher. The KEYNOTE-826 trial (Colombo et al., 2021) presented further evidence that the addition of pembrolizumab to chemotherapy, with or without bevacizumab, could significantly enhance the progression-free survival (PFS) and overall survival (OS) in patients with persistent, recurrent, or metastatic cervical cancer. The greatest improvement appears to be observed in patients with a PD-L1 CPS of 1 or higher. According to the National Comprehensive Center Network (NCCN) Guideline version 2022, pembrolizumab in combination with cisplatin and paclitaxel has been recommended as a first-line treatment for PD-L1-positive patients with recurrent or metastatic cervical cancer.

Similar to pembrolizumab, sintilimab is a fully human IgG4 monoclonal antibody that binds to PD-1, thereby inhibiting the interaction between PD-1 and its ligands (PD-L1 and PD-L2) (Lu et al., 2022a). Sintilimab was initially approved for the treatment of relapsed or refractory patients with classical Hodgkin lymphoma who had received two or more lines of systemic chemotherapy (Hoy, 2019). Subsequently, the efficacy and safety of sintilimab was validated in other advanced or metastatic solid tumors, including esophageal squamous cell carcinoma (Lu et al., 2022b) and non-small cell lung cancer (NSCLC) (Yang et al., 2021; Zhou et al., 2021). Nevertheless, there is limited information available regarding the utility and toxicity of sintilimab in cervical cancer. A prospective phase II trial (Xu et al., 2022) investigated the effectiveness of sintilimab in combination with anlotinib for PD-L1-positive patients with recurrent or metastatic cervical cancer. The study showed that patients with mutations in PIK3CA, PI3K-AKT signaling, or KMT2D achieved a higher ORR, suggesting the presence of other biomarkers related to efficacy in patients with PD-L1-positive recurrent or metastatic cervical cancer. Moreover, several trials have demonstrated the efficacy of some PD-1 monoclonal antibodies in the treatment of these cancers. However, the discrepancy in efficacy of PD-1 inhibitors between PD-L1-positive and PD-L1-negative patients remains unconfirmed. For instance, the ORRs of socazolimab in patients with a CPS ≥1 and <1 were 16.7% and 17.9%, respectively (An et al., 2022), while the rates for cemiplimab were 18% and 11%, respectively (Tewari et al., 2022). The current study evaluated the efficacy and safety of sintilimab, with or without adjuvant therapy, as a first-line or second-line therapy for patients with recurrent or metastatic cervical cancer, regardless of PD-L1 status.

## Materials & Methods

### Patients

Twenty-seven patients with recurrent or metastatic cervical cancer were enrolled in the study regardless of whether they previously received bevacizumab or chemotherapy. This study received approval from the Ethics Committee of the First Affiliated Hospital of the Army Medical University, PLA, with a waiver of informed consent (approval number: [B]KY2023007). The eligibility criteria was a histologically or radiologically confirmed recurrent or metastatic cervical cancer, with at least one measurable disease according to the Response Evaluation Criteria in Solid Tumors version 1.1 (RECIST v1.1). PD-L1 was not assessed in any of the patients, as samples were unavailable in some cases and financial constraints prevented testing in others. Patients who had previously received other immune checkpoint inhibitors were excluded.

### Treatment

Patients were administered sintilimab at a dose of 200 mg intravenously once every three weeks, either as a monotherapy or in combination with chemotherapy. Chemotherapy was used as a combination regimen for patients who had not received systemic chemotherapy, unless they refused or were unable to tolerate the treatment. Relevant laboratory tests and examinations were performed prior to each treatment. In this study, the efficacy of four patients could not be evaluated, as they did not undergo imaging assessments following sintilimab treatment. The expected withdrawal time for sintilimab is two years. Treatment delays or discontinuations caused by intolerable adverse events (AEs) and other practical reasons were permitted.

### Assessments

The primary metric in this analysis was ORR, which included the proportion of patients with complete responses (CR) and partial responses (PR). Tumor response was assessed following the guidelines of RECIST v1.1. Adverse events of any grade were recorded from the initiation of sintilimab therapy until the end of the follow-up period. Overall survival (OS) was defined as the duration in months from the first dose of sintilimab to death or the last follow-up.

### Statistical analysis

This study used the Clopper–Pearson method to estimate the ORR and calculate the corresponding 95% confidence interval (CI). The efficacy comparisons among different histologies, therapy methods, and treatment cycles were performed using the chi-square test. The Kaplan–Meier curves and log-rank tests were conducted using R version 4.2.1.

## Results

### Characteristics of patients

Between November 2019 and July 2022, 27 patients were enrolled in the study (intention-to-treat population [ITT] population). The median patient age was 54 years old (range, 41 to 69 years). In the primary treatment phase, 25 patients received surgery, with 24 patients undergoing radical hysterectomy and one patient undergoing total hysterectomy. The remaining two patients received chemoradiotherapy. Among the patients who underwent surgery, eight out of 25 (32.0%) received neoadjuvant chemotherapy before surgery, while all 25 patients (100%) received adjuvant platinum-based chemotherapy, and 20 out of 25 patients (80%) received adjuvant radiotherapy following the surgery.

Regarding histology, 23 out of 27 patients (85.2%) had squamous cell carcinoma (SCC), while only four (14.8%) patients had adenocarcinoma, including one patient with a confirmed microsatellite instability-high (MSI-H) tumor. The median time from the end of primary treatment to recurrence or metastasis was 20 months (range, 5–117 months). Prior to receiving sintilimab treatment, eight out of 27 patients (29.6%) had previously undergone platinum-based chemotherapy regimens after recurrence or metastasis, among which, one patient received bevacizumab, and two patients received concurrent radiotherapy. The median time from recurrence or metastasis to sintilimab treatment was two months, ranging from one to 20 months. During the course of sintilimab treatment, 20 patients (74.1%) received combination therapy, and seven patients (25.9%) received monotherapy (Table 1).

**Table 1:** Baseline characteristics (*N* = 27).

Characteristic	No. (%)
Age, median (range)	54 (41–69)
Histology	
Squamous cell carcinoma (SCC)	23 (85.2)
Adenocarcinoma	4 (14.8)
FIGO (2018) stage	
IB	5 (18.5)
IIA	7 (25.9)
IIB	4 (14.8)
IIIA	1 (3.7)
IIIC	10 (37.0)
Therapy before sintilimab treatment	
Platinum-based chemotherapy	5 (18.5)
Platinum-based chemotherapy plus bevacizumab	1 (3.7)
Platinum-based chemotherapy plus radiotherapy	2 (7.4)
None	19 (70.4)
Status of sintilimab treatment	
First-line treatment	23 (85.2)
Second-line treatment	4 (14.8)
Therapy during sintilimab treatment	
Cisplatin/carboplatin plus paclitaxel	17 (63.0)
Bevacizumab	2 (7.4)
Radiotherapy	1 (3.7)
None	7 (25.9)

**Figure 1:**
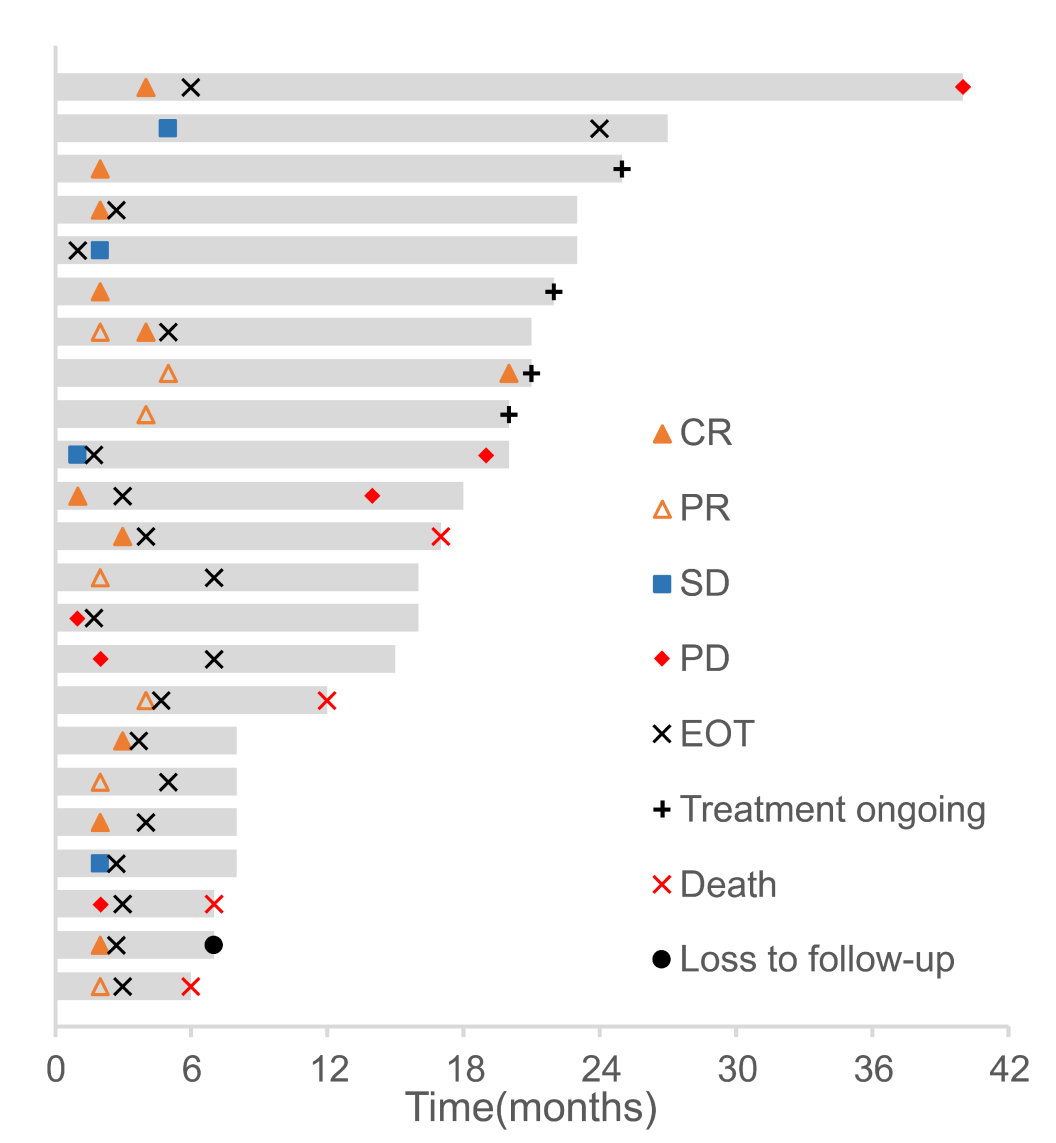
Responses of patients in the efficacy-evaluable population. The duration of follow-up of each patient is exhibited by the length of each bar. Abbreviations: CR, complete response; PD, progressive disease; PR, partial response; SD, stable disease; EOT, end of treatment.

At the last follow-up in March 2023, four out of 27 (14.8%) patients had remained on sintilimab treatment, while 23 patients (85.2%) had discontinued treatment, including three patients (11.1%) due to progressive disease (PD) and five patients (18.5%) due to AEs. Six patients (22.2%) discontinued therapy due to CR, which they had achieved before completing the full course of sintilimab treatment. Of these, two patients who discontinued treatment after receiving four and nine cycles of sintilimab, respectively, eventually progressed to PD after withdrawal of therapy (Fig. 1).

### Efficacy evaluation

**Figure 2:**
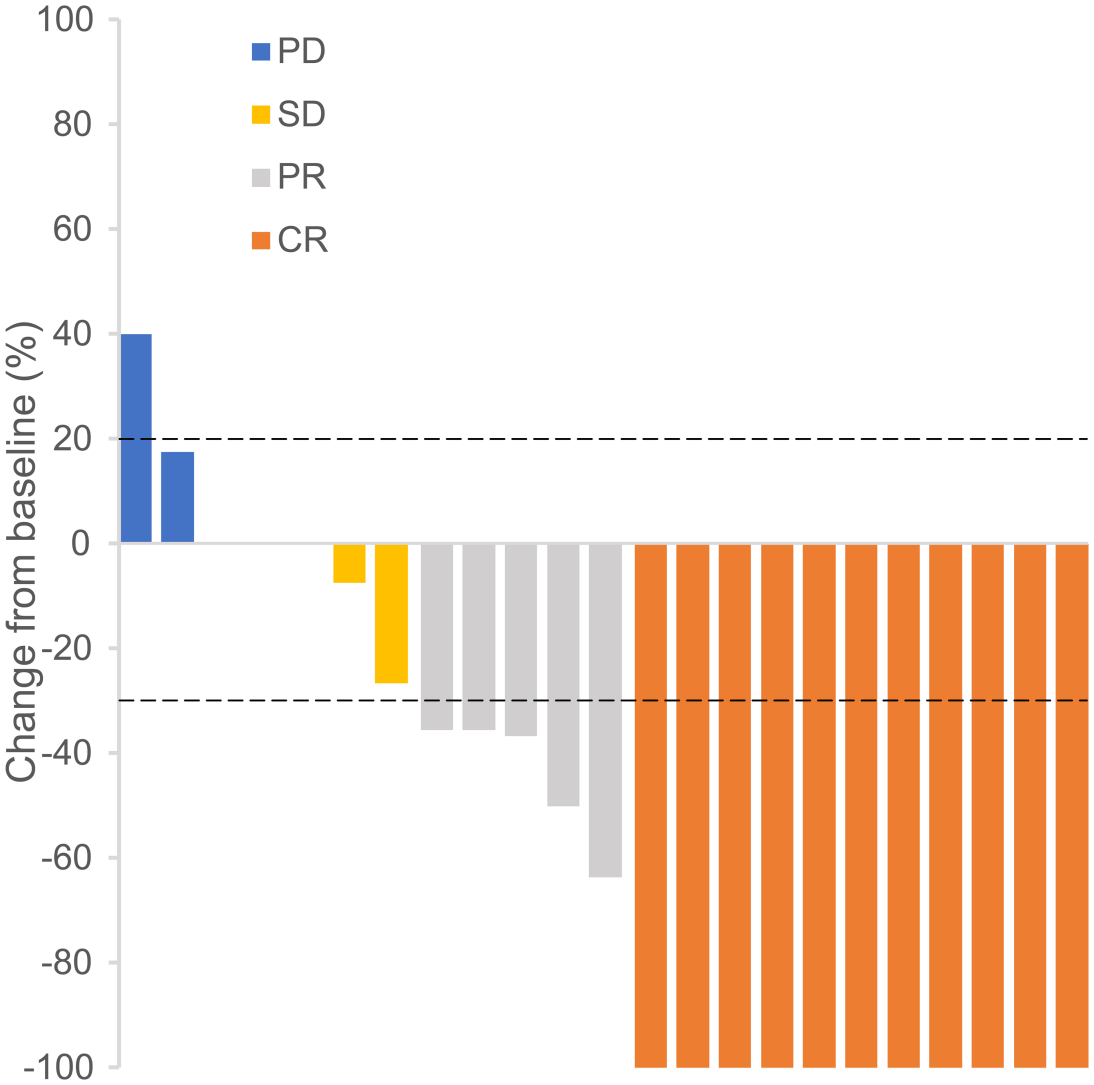
Changes from baseline of targeted tumor size in each patient of the efficacy-evaluable population. Abbreviations: CR, complete response; PD, progressive disease; PR, partial response; SD, stable disease.

For the ITT population, the median OS was 17 months (range, 6–40 months). Four patients were categorized into the efficacy-unevaluable population due to the lack of timely evaluation after they received sintilimab. Finally, 23 patients were enrolled in the efficacy-evaluable population (Table 2). Eleven patients achieved CR and five patients attained PR. One patient with CR and two patients with PR died due to discontinuation of treatment because of AEs. Four patients achieved stable disease (SD) and three patients experienced PD, and two out of the three PD patients developed new lesions during the course of treatment (Fig. 2). One patient with SD requested to switch to radiotherapy after receiving only one cycle of sintilimab. Among the three patients who experienced PD, two switched to radiotherapy and the remaining patient died. The confirmed ORR and disease control rate (DCR) were 59.3% (95% CI [38.8–77.6]) and 74.1% (95% CI [53.7–88.9]), respectively, in the ITT population and 69.6% (95% CI [47.1–86.8]) and 87.0% (95% CI [66.4–97.2]), respectively, in the efficacy-evaluable population (Table 2).

**Table 2:** Efficacy evaluation.

Efficacy	ITT population (*N* = 27)	Efficacy-evaluable population (*N* = 23)
ORR	16 (59.3)	16 (69.6)
95% CI	38.8 to 77.6	47.1 to 86.8
DCR	20 (74.1)	20 (87.0)
95% CI	53.7 to 88.9	66.4 to 97.2
CR	11 (40.7)	11 (47.8)
PR	5 (18.5)	5 (21.7)
SD	4 (14.8)	4 (17.4)
PD	3 (11.1)	3 (13.0)
Unknown	4 (14.8)	–

**Notes.**

Abbreviations CI confidence interval CR complete response DCR disease control rate ITT intention-to-treat ORR objective response rate PD progressive disease PR partial response SD stable disease

In the efficacy-evaluable population, the median number of treatment cycles was six. No significant difference was observed in the ORR of patients with ≤6 and >6 cycles of treatment (57.1% *vs.* 88.9%, *P* = 0.176; Table S1), nor was a significant difference observed in the ORR of patients with different histological types (75.0% *vs.* 33.3%, *P* = 0.209; Table S2). Objective response was achieved in 15 of 20 SCC patients and one of three adenocarcinoma patients. Notably, the patient with adenocarcinoma who achieved objective response was found to have MSI-H. The ORR in patients with combination therapy was significantly higher than that in patients with monotherapy; specifically, the ORRs were 82.3% and 33.3% in patients who received sintilimab with or without adjuvant therapy, respectively (*P* = 0.045; Table S3). No significant difference was observed in ORRs between patients receiving sintilimab as a first-line treatment and those receiving sintilimab as a second-line treatment, with ORRs being 70.0% and 66.7%, respectively (*P* = 1.000; Table S4). For patients receiving sintilimab as first-line therapy, the ORR was significantly higher in patients who started sintilimab treatment within two months of recurrence or metastasis compared to those who started sintilimab treatment after two months (91.7% *vs* 37.5%, *P* = 0.018; Table S5).

**Figure 3:**
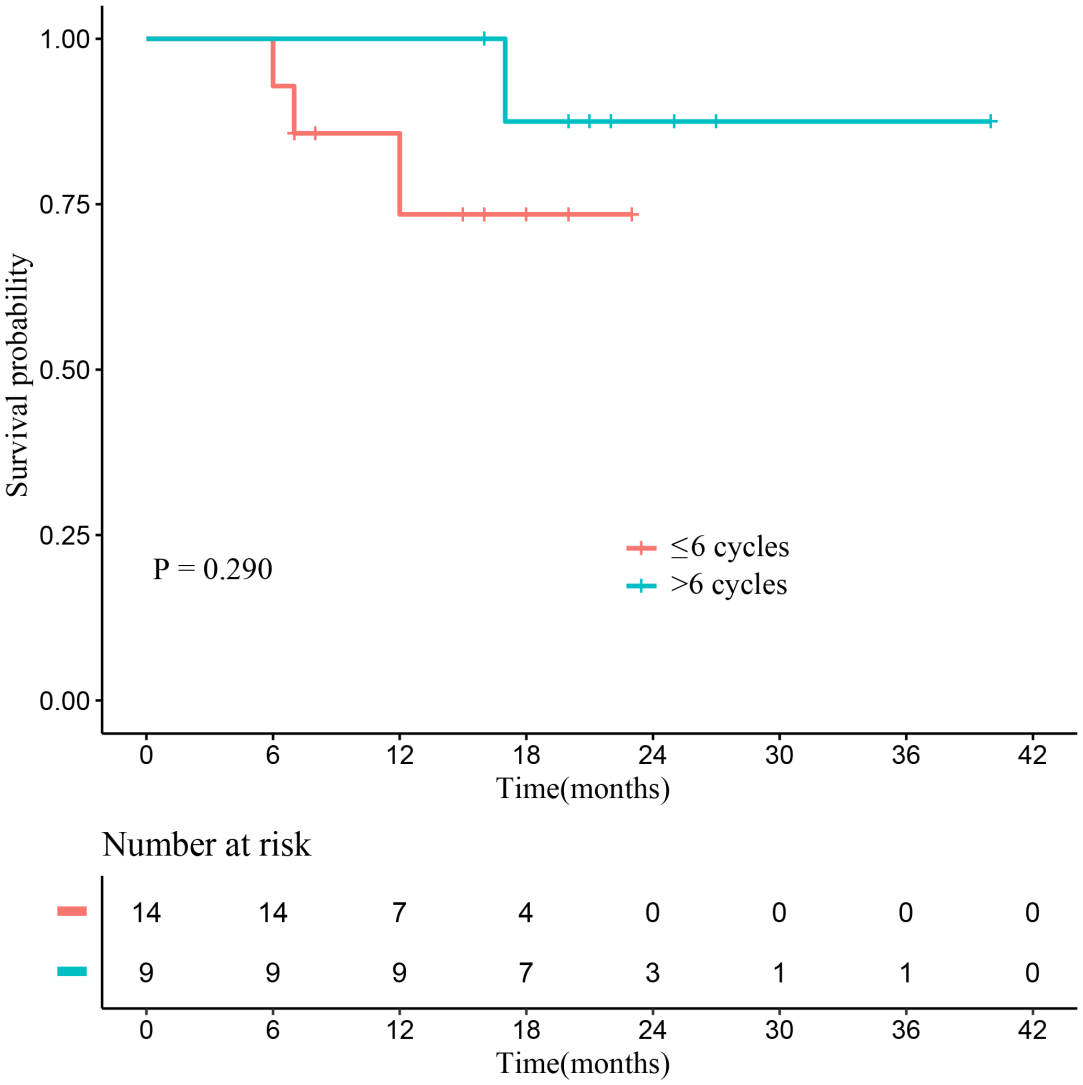
The Kaplan–Meier curves of OS of the efficacy-unevaluable population stratified by cycles of sintilimab (≤ 6 cycles *vs.*> 6 cycles). Abbreviations: OS, overall survival.

**Figure 4:**
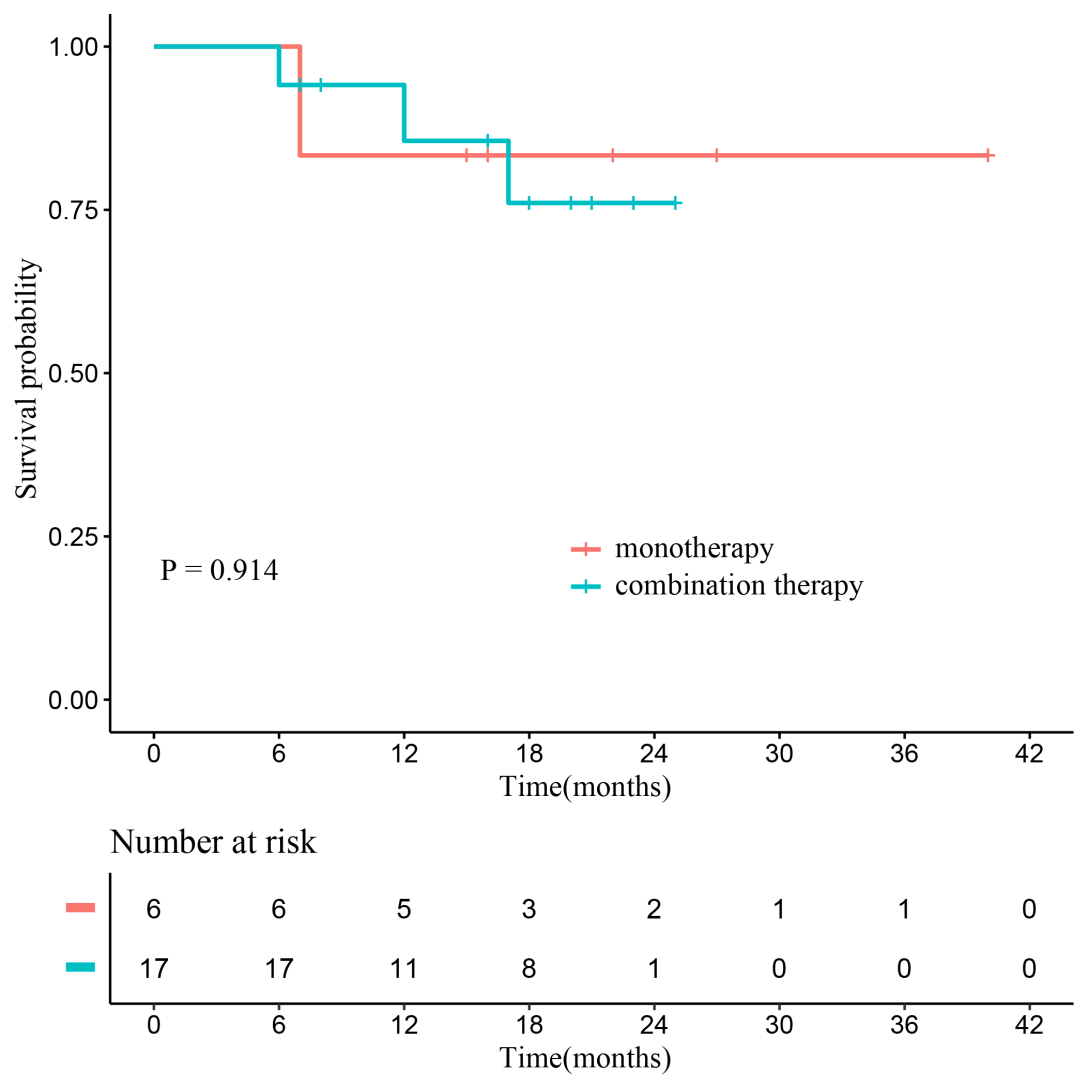
The Kaplan–Meier curves of OS in the efficacy-unevaluable population stratified by therapy regimens (monotherapy *vs.* combination therapy). Abbreviations: OS, overall survival.

As of the study date cutoff, five patients (18.5%) had died, comprising one patient with PD, three patients who achieved objective response but discontinued treatment due to AEs, and one patient in the efficacy-unevaluable population. No significant difference in OS was found between patients who received ≤6 cycles and those who received >6 cycles of sintilimab (*P* = 0.290; Fig. 3). Additionally, there was also no notable difference in OS between patients who received monotherapy and those who received combination therapy (*P* = 0.914; Fig. 4).

### Safety

All adverse events (Table 3) were evaluated using the Common Terminology Criteria for Adverse Events (CTCAE) Version 5.0. A total of 15 patients (55.5%) experienced one or more treatment-related AEs, but no patient experienced grade 5 AEs. The most common AEs were nausea (25.9%), vomiting (25.9%), and decreased white blood cell count (22.2%). The most severe AE observed was gamma-glutamyl transferase (GGT) elevation, which occurred in one patient. Grade 3 AEs were reported in four (14.8%) patients. These four patients experienced concurrent symptoms of nausea, vomiting, and weight loss, with one patient also experiencing hypokalemia. Five patients (18.5%) discontinued treatment due to AEs, including four patients with severe digestive symptoms and one patient with hypothyroidism.

**Table 3:** AEs.

AEs (*N* = 27)	Total, N (%)	N (%)			
		Grade 1	Grade 2	Grade 3	Grade 4
Anemia	3 (11.1)	0 (0)	3 (11.1)	0 (0)	0 (0)
Platelet count decreased	2 (7.4)	1 (3.7)	1 (3.7)	0 (0)	0 (0)
White blood cell decreased	6 (22.2)	2 (7.4)	4 (14.8)	0 (0)	0 (0)
Nausea	7 (25.9)	3 (11.1)	0 (0)	4 (14.8)	0 (0)
Vomiting	7 (25.9)	3 (11.1)	0 (0)	4 (14.8)	0 (0)
Stomach pain	2 (7.4)	2 (7.4)	0 (0)	0 (0)	0 (0)
Diarrhea	2 (7.4)	2 (7.4)	0 (0)	0 (0)	0 (0)
Weight loss	4 (14.8)	0 (0)	0 (0)	4 (14.8)	0 (0)
Weight gain	1 (3.7)	0 (0)	1 (3.7)	0 (0)	0 (0)
ALT increased	5 (18.5)	4 (14.8)	1 (3.7)	0 (0)	0 (0)
AST increased	5 (18.5)	3 (11.1)	2 (7.4)	0 (0)	0 (0)
GGT increased	3 (11.1)	1 (3.7)	1 (3.7)	0 (0)	1 (3.7)
Hypokalemia	3 (11.1)	2 (7.4)	0 (0)	1 (3.7)	0 (0)
Hypothyroidism	2 (7.4)	0 (0)	2 (7.4)	0 (0)	0 (0)
AEs resulting in discontinued treatment	5 (18.5)	0 (0)	1 (3.7)	4 (14.8)	0 (0)

**Notes.**

Abbreviations AEs adverse events ALT alanine aminotransferase increased AST aspartate aminotransferase GGT gamma-glutamyl transferase

## Discussion

The potential efficacy of PD-1 monoclonal antibodies for cervical cancer has been explored in various clinical trials, especially in the second-line treatment of recurrent or metastatic cervical cancer. There is, however, limited research on the benefit of PD-1 monoclonal antibodies as a first-line treatment for recurrent or metastatic cervical cancer. In this study, the confirmed ORR was 69.6% (16 of 23) in the efficacy-evaluable population. The ORR was 70.0% (14 of 20) for patients receiving sintilimab as first-line therapy and 66.7% (2 of 3) for those receiving it as second-line therapy. The effectiveness of sintilimab was demonstrated in both first-line and second-line therapies for recurrent or metastatic cervical cancer. Pembrolizumab has been extensively investigated in the treatment of recurrent or metastatic cervical cancer. In the KEYNOTE-158 study (Chung et al., 2019), which enrolled 82 patients with PD-L1-positive disease, pembrolizumab demonstrated an ORR of 12.2% and a DCR of 30.6%. In PD-L1-positive patients, pembrolizumab showed an ORR of 14.6% and a DCR of 32.9%. However, in PD-L1-negative patients, the ORR was 0%, and the DCR was 20%. In addition to pembrolizumab, other PD-1 monoclonal antibodies have been investigated in recurrent or metastatic patients who progressed after one or more lines of systemic therapy. In the CheckMate 358 trial (Naumann et al., 2019), which included 10 patients with PD-L1-positive disease, nivolumab monotherapy (240 mg/2 weeks) achieved an ORR of 26.3% and a DCR of 68.4%. Meanwhile, nivolumab monotherapy (three mg/kg/2 weeks) only achieved an ORR of 4% in another trial in which 17(68%) of 25 patients had a PD-L1-positive tumor (Santin et al., 2020). Thus, the utility of nivolumab was variable across different trials. In another trial involving 91 patients, with 54 (59.3%) patients being CPS ≥1, socazolimab monotherapy yielded an ORR of 15.4% and a DCR of 49.5%. The ORRs among patients with CPS ≥1 and <1 were 16.7% and 17.9%, respectively (An et al., 2022). In a clinical trial comparing the efficacy of cemiplimab and chemotherapy in recurrent or metastatic cervical carcinoma, the ORR was found to be higher in the cemiplimab group compared to the chemotherapy group (16.4% *vs.* 6.3%, *P* < 0.001). Furthermore, when analyzing the cemiplimab monotherapy subgroup, the ORRs observed in patients with PD-L1 expression ≥1% and <1% were 18% and 11%, respectively (Tewari et al., 2022). These clinical trials indicated that PD-1 monoclonal antibodies might provide a superior ORR compared to chemotherapy in the second line treatment of recurrent and metastatic cervical cancer.

Given the limitation of the efficacy of PD-1 monoclonal antibody monotherapy and the expectation to improve outcomes of patients with recurrent or metastatic cervical cancer, several combination therapy regimens have been explored. In the KEYNOTE-826 trial (Colombo et al., 2021), where 88.6% of the enrolled patients had a PD-L1 CPS ≥1, the combination of pembrolizumab and platinum-based chemotherapy demonstrated an ORR of 65.9%, and a DCR of 88.3%. Among patients with a PD-L1 CPS ≥1, the ORR and DCR were 68.1% and 89.3%, respectively, while for patients with a PD-L1 CPS <1, the ORR and DCR were 48.6% and 80%, respectively. In addition to investigating the combination of PD-1 monoclonal antibodies with chemotherapy, efforts have been made to evaluate the effectiveness of combining PD-1 monoclonal antibodies with another targeted therapy for the treatment of recurrent or metastatic cervical cancer. Balstilimab, combined with zalifrelimab, targeting PD-1 and anticytotoxic T-lymphocyte–associated antigen-4 (CTLA-4), respectively, attained an ORR of 25.6% and a DCR of 52% as a regimen for second-line treatment for recurrent or metastatic cervical cancer (O’Malley et al., 2022). The ORRs in PD-L1-positive and PD-L1-negative patients were 32.8% and 9.1%, respectively. The CLAP study (Lan et al., 2020) reported an ORR of 55.6% and a DCR of 82.2% for camrelizumab (a PD-1 monoclonal antibody) combined with apatinib (a tyrosine kinase inhibitor) in patients with metastatic, recurrent, or persistent cervical cancer. In the CLAP study, where 66.7% of the enrolled patients had PD-L1-positive disease, the ORRs were 69.0% and 50.0% for patients with PD-L1-positive and PD-L1-negative disease, respectively (*P* = 0.281). A regimen of sintilimab and anlotinib, a multikinase inhibitor, achieved an ORR of 54.8% and a DCR of 88.1% in a phase II trial where all enrolled patients had PD-L1-positive recurrent or metastatic cervical cancer (Xu et al., 2022). Compared to monotherapy with PD-1 monoclonal antibodies, combination therapy appeared to exhibit more effective antitumor activities. A similar result was observed in the current study, where the ORR of the combination therapy was significantly higher than that of sintilimab monotherapy (82.3% *vs.* 33.3%, *P* = 0.045).

Previous research showed that the expression of PD-L1 was significantly increased in cervical intraepithelial neoplasia (CIN) and cervical squamous cell cancer compared to normal cervical epithelia (Mezache et al., 2015). Furthermore, the expression level of PD-1 and PD-L1 was positively correlated with the progression of CIN and the metastasis of squamous cell carcinoma (Yang et al., 2017). PD-L1 expression could potentially serve as a therapeutic target and a prognostic factor for cervical cancer. The antitumor effectiveness and safety of PD-1 monoclonal antibodies was first investigated in PD-L1-positive cervical cancer patients in the KEYNOTE-028 trial (Frenel et al., 2017). In the follow-up KEYNOTE-158 study (Chung et al., 2019), it was observed that all objective responses to pembrolizumab occurred in patients with PD-L1-positive recurrent or metastatic cervical cancer. This result suggests that pembrolizumab exhibits better antitumor activity in patients with PD-L1-positive recurrent or metastatic cervical cancer, compared to those with PD-L1-negative status. However, a similar efficacy was observed for several PD-1 monoclonal antibodies in patients with PD-L1-negative tumors. PD-L1 expression has been found to be correlated with many factors including age (Feng et al., 2018), HPV infection, parity and abortion (Feng et al., 2018), histology (Heeren et al., 2016), and chemotherapy history (Meng et al., 2018). In addition, PD-L1 immunohistochemistry assays with different sensitivities in different trials might contribute to discrepancies in PD-L1 expression levels observed among various studies. In summary, whether there is significant difference in the efficacy of PD-1 monoclonal antibodies between patients with PD-L1-positive and PD-L1-negative cervical cancer remains to be substantiated. Notably, the detection of PD-L1 is not always convenient in certain hospital environments and might also be refused by patients. Additionally, there may be unknown biomarkers influencing treatment efficacy. Thus, it is reasonable to explore the efficacy of immune checkpoint inhibitors in patients with recurrent and metastatic cervical cancer, regardless of their PD-L1 status. In the current study, the confirmed ORR and DCR were 59.3% (95% CI [38.8–77.6]) and 74.1% (95% CI [53.7–88.9]), respectively, in the intention-to-treat population and were 69.6% (95% CI [47.1–86.8]) and 87.0% (95% CI [66.4–97.2]), respectively, in the efficacy-evaluable population. Therefore, the use of PD-1 inhibitors in all recurrent or metastatic patients regardless of PD-L1 expression appears to be a reasonable approach.

Moreover, patients who initiated sintilimab treatment within two months of recurrence as a first-line therapy showed a notably higher ORR than those who commenced treatment more than two months after recurrence (91.7% *vs.* 37.5%, *P* = 0.018). This suggests that initiating PD-1 therapy within two months of recurrence is linked to better treatment outcomes. However, it must be noted that the sample size of the current study was small, and some patients received insufficient immunotherapy. It is important to acknowledge that comparisons between different studies are limited due to variations in prior systemic therapy and PD-L1 expression. Well-designed clinical trials are necessary in the future to validate these findings.

The safety profile of sintilimab combined with chemotherapy in this study was consistent with previous reports of patients with NSCLC and esophageal squamous cell carcinoma. No grade 5 AEs were observed. AEs occurred in 55.5% of patients, but only 18.5% of patients experienced grade 3 or 4 AEs. The most common AEs, such as nausea, vomiting, and weight loss, might be related to chemotherapy. Discontinuation of treatment due to AEs was observed in five patients (18.5%). Among them, only one patient was diagnosed with hypothyroidism that was potentially associated with sintilimab. No instances of rash, immune myocarditis, immune pneumonitis, or fistula were reported during the study period.

## Conclusions

In conclusion, this study demonstrated the promising efficacy and manageable toxicity of sintilimab as a monotherapy or in combination with adjuvant therapy for recurrent or metastatic cervical cancer. Furthermore, early initiation of sintilimab as first-line therapy within two months following recurrence is more effective compared to delayed initiation beyond this period in patients with recurrent disease. However, several limitations should be acknowledged. First, this was a retrospective study, which may introduce selection bias. Second, PD-L1 status, which might impact patient response and prognosis, was not established. Additionally, the small sample size and high rate of treatment discontinuation unrelated to adverse events or efficacy may have reduced the certainty and precision of the results. Therefore, larger-scale randomized controlled trials are required to further investigate the potential benefits of sintilimab in this patient population.

##  Supplemental Information

10.7717/peerj.20477/supp-1 Supplemental Information 1 Efficacy evaluation of different cycles in the efficacy-evaluable population Abbreviations: CI, confidence interval; CR, complete response; ORR, objective response rate; PD, progressive disease; PR, partial response; SD, stable disease.

10.7717/peerj.20477/supp-2 Supplemental Information 2 Efficacy evaluation of different histological types in the efficacy-evaluable population Abbreviations: CI, confidence interval; CR, complete response; ORR, objective response rate; PD, progressive disease; PR, partial response; SCC, Squamous cell carcinoma; SD, stable disease.

10.7717/peerj.20477/supp-3 Supplemental Information 3 Efficacy evaluation of different therapy methods in the efficacy-evaluable population Abbreviations: CI, confidence interval; CR, complete response; ORR, objective response rate; PD, progressive disease; PR, partial response; SD, stable disease.

10.7717/peerj.20477/supp-4 Supplemental Information 4 Efficacy evaluation of first-line and second-line therapy in the efficacy-evaluable population Abbreviations: CI, confidence interval; CR, complete response; ORR, objective response rate; PD, progressive disease; PR, partial response; SD, stable disease.

10.7717/peerj.20477/supp-5 Supplemental Information 5 In the first-line therapy population of the efficacy-evaluable population, the efficacy evaluation of patients who started sintilimab treatment at different times Abbreviations: CI, confidence interval; CR, complete response; ORR, objective response rate; PD, progressive disease; PR, partial response; SD, stable disease.

10.7717/peerj.20477/supp-6 Supplemental Information 6 Raw Data The characteristics of patients, efficacy evaluation and safety of cervical patients who received PD-1 inhibitor.

## Additional Information and Declarations

### Data Availability

The following information was supplied regarding data availability: The raw data is available in the Supplementary File.
