# Supplementary material for: Real-world efficacy assessment for sintilimab in recurrent or metastatic cervical cancer
Source: PeerJ. 2025 Dec 19;13:e20477. doi: 10.7717/peerj.20477 (PMC12721100; doi:10.7717/peerj.20477)
Supplement: Supplemental Information 1 — Abbreviations: CI, confidence interval; CR, complete response; ORR, objective response rate; PD, progressive disease; PR, partial response; SD, stable disease. [file peerj-13-20477-s001.docx]

Supplementary Table 1. Efficacy evaluation of different cycles in the efficacy-evaluable population.

| Efficacy (N=23) | ≤6 cycles (N=14) | >6 cycles (N=9) | P-value |
| --- | --- | --- | --- |
| ORR | 8(57.1) | 8(88.9) | 0.176 |
| 95% CI | 28.9 to 82.3 | 51.8 to 99.7 |  |
| CR | 5(35.7) | 6(66.7) | 0.214 |
| PR | 3(21.4) | 2(22.2) | 1.000 |
| SD | 3(21.4) | 1(11.1) | 1.000 |
| PD | 3(21.4) | 0(0) | 0.253 |

Abbreviations: CI, confidence interval; CR, complete response; ORR, objective response rate; PD, progressive disease; PR, partial response; SD, stable disease.
