# Supplementary material for: Real-world efficacy assessment for sintilimab in recurrent or metastatic cervical cancer
Source: PeerJ. 2025 Dec 19;13:e20477. doi: 10.7717/peerj.20477 (PMC12721100; doi:10.7717/peerj.20477)
Supplement: Supplemental Information 2 — Abbreviations: CI, confidence interval; CR, complete response; ORR, objective response rate; PD, progressive disease; PR, partial response; SCC, Squamous cell carcinoma; SD, stable disease. [file peerj-13-20477-s002.docx]

Supplementary Table 2. Efficacy evaluation of different histological types in the efficacy-evaluable population.

| Efficacy (N=23) | SCC (N=20) | Adenocarcinoma (N=3) | *P-*value |
| --- | --- | --- | --- |
| ORR | 15(75.0) | 1(33.3) | 0.209 |
| 95% CI | 50.9 to 91.3 | 0.8 to 90.6 |  |
| CR | 11(55.5) | 0(0) | 0.217 |
| PR | 4(20.0) | 1(33.3) | 0.539 |
| SD | 3(15.0) | 1(33.3) | 0.453 |
| PD | 2(10.0) | 1(33.3) | 0.356 |

Abbreviations: CI, confidence interval; CR, complete response; ORR, objective response rate; PD, progressive disease; PR, partial response; SCC, Squamous cell carcinoma; SD, stable disease.
