# Supplementary material for: Real-world efficacy assessment for sintilimab in recurrent or metastatic cervical cancer
Source: PeerJ. 2025 Dec 19;13:e20477. doi: 10.7717/peerj.20477 (PMC12721100; doi:10.7717/peerj.20477)
Supplement: Supplemental Information 3 — Abbreviations: CI, confidence interval; CR, complete response; ORR, objective response rate; PD, progressive disease; PR, partial response; SD, stable disease. [file peerj-13-20477-s003.docx]

Supplementary Table 3. Efficacy evaluation of different therapy methods in the efficacy-evaluable population.

| Efficacy (N=23) | monotherapy (N=6) | Combination therapy(N=17) | *P-*value |
| --- | --- | --- | --- |
| ORR | 2(33.3) | 14 (82.3) | 0.045 |
| 95% CI | 4.3 to 77.7 | 56.6 to 96.2 |  |
| CR | 2(33.3) | 9(52.9) | 0.640 |
| PR | 0(0) | 5(29.4) | 0.272 |
| SD | 1(16.7) | 3(17.6) | 1.000 |
| PD | 3(50.0) | 0(0) | 0.011 |

Abbreviations: CI, confidence interval; CR, complete response; ORR, objective response rate; PD, progressive disease; PR, partial response; SD, stable disease.
