# Supplementary material for: Real-world efficacy assessment for sintilimab in recurrent or metastatic cervical cancer
Source: PeerJ. 2025 Dec 19;13:e20477. doi: 10.7717/peerj.20477 (PMC12721100; doi:10.7717/peerj.20477)
Supplement: Supplemental Information 4 — Abbreviations: CI, confidence interval; CR, complete response; ORR, objective response rate; PD, progressive disease; PR, partial response; SD, stable disease. [file peerj-13-20477-s004.docx]

Supplementary Table 4. Efficacy evaluation of first-line and second-line therapy in the efficacy-evaluable population.

| Efficacy (N=23) | First-line (N=20) | Second-line (N=3) | *P-*value |
| --- | --- | --- | --- |
| ORR | 14(70.0) | 2(66.7) | 1.000 |
| 95% CI | 45.7 to 88.1 | 9.4 to 99.2 |  |
| CR | 10(50.0) | 1(33.3) | 1.000 |
| PR | 4(20.0) | 1(33.3) | 0.539 |
| SD | 4(20.0) | 0(0) | 1.000 |
| PD | 2(10.0) | 1(33.3) | 0.356 |

Abbreviations: CI, confidence interval; CR, complete response; ORR, objective response rate; PD, progressive disease; PR, partial response; SD, stable disease.
