# Supplementary material for: Real-world efficacy assessment for sintilimab in recurrent or metastatic cervical cancer
Source: PeerJ. 2025 Dec 19;13:e20477. doi: 10.7717/peerj.20477 (PMC12721100; doi:10.7717/peerj.20477)
Supplement: Supplemental Information 5 — Abbreviations: CI, confidence interval; CR, complete response; ORR, objective response rate; PD, progressive disease; PR, partial response; SD, stable disease. [file peerj-13-20477-s005.docx]

Supplementary Table 5. In the first-line therapy population of the efficacy-evaluable population, the efficacy evaluation of patients who started sintilimab treatment at different times.

| Efficacy (N=20) | ≤2 months (N=12) | >2 months (N=8) | *P-*value |
| --- | --- | --- | --- |
| ORR | 11(91.7) | 3(37.5) | 0.018 |
| 95% CI | 61.5 to 99.8 | 8.5 to 75.5 |  |
| CR | 7(58.3) | 3(37.5) | 0.650 |
| PR | 4(33.3) | 0(0) | 0.117 |
| SD | 0(0) | 4(50.0) | 0.014 |
| PD | 1(8.3) | 1(12.5) | 1.000 |

Abbreviations: CI, confidence interval; CR, complete response; ORR, objective response rate; PD, progressive disease; PR, partial response; SD, stable disease.
